# 30 years of CIK cell therapy: recapitulating the key breakthroughs and future perspective

**DOI:** 10.1186/s13046-021-02184-2

**Published:** 2021-12-09

**Authors:** Amit Sharma, Ingo G. H. Schmidt-Wolf

**Affiliations:** 1 Department of Integrated Oncology, Center for Integrated Oncology (CIO), University Hospital Bonn, 53127 Bonn, Germany; 2 Department of Neurosurgery, University Hospital Bonn, 53127 Bonn, Germany

**Keywords:** Cytokine-induced killer (CIK) cell therapy, Immunotherapies

## Abstract

Emerging evidence from the numerous clinical trials involving cytokine-induced killer (CIK) cell therapy suggests that its optimization in combination with other contemporary cancer therapies in a complementary manner (rather than as competition) will be a key to combat cancer.

## Main text

Beyond traditional cancer therapies, cancer immunotherapy has become a mainstay and revolutionized the way cancer is treated. In fact, the combination treatments involving immunotherapies has been shown to be more favorable in terms of survival and reduced side effects. Within this spectrum, the cytokine-induced killer (CIK) cell therapy holds a special place. This is evident from the several (> 80) clinical trials involving CIK cells ranging from solid tumors to malignant blood diseases (clinicaltrial.gov). Since its introduction in 1991 [1], more than 5000 patients with over 30 different tumor types have been treated in clinical trials with CIK cells alone or in combination with supportive therapies to date [2]. Moreover, 27 CIK-based studies reported significant improvement in median progression-free survival and overall survival, while 9 studies reported significantly prolongation of 5-year survival. Meanwhile, CIK cell treatment is licensed in many countries, including Germany. It must be acknowledged that CIK therapy has the advantage over other cancer immunotherapies as it eliminates cancer cells by transfusing immune cells that have been expanded and activated in vitro [3]. Besides, being inexpensive, CIK cells are also easier to expand and readily reach the clinically relevant doses with standard culturing protocols. While its contemporaries such primary NK cells have limited proliferative capacity and usually rely on accessory cells (feeder cells) in order to achieve a robust yield. It is also worth mentioning that CIK cells, as a heterogeneous cell population, possess the ability to attack a variety of tumor targets, contrary to other immunotherapeutic approaches. Also, CIK cells exhibit the ability for MHC-unrestricted targeting of tumors and for reduced alloreactivity across MHC barriers. Besides, the convenient method to generate and culture CIK cell also offers the possibility to enhance their cytotoxic potential within the controlled experimental framework. Of note, the dendritic cell (DC) co-culture with CIK cells have shown significant increase in the antitumor immunity and enhanced cytotoxic activity compared to CIK cell treatment alone [4]. As an extended version of the CIK approach, DC-CIK therapy also paves the way for targeting a range of malignancies.

Ever since it was observed that most of the cytotoxicity of CIK cells resulted from the interactions with members of the natural killer group 2 (NKG2D) [5, 6], there have been excitement about the distant role of CIK cells towards tumor immunosurveillance. To expand the horizon beyond the pivotal role of NKG2D in CIK cell-mediated cytotoxicity, the additional axis of ADCC (via CD16 or other yet to be known factors) requires further attention. On broader prospective, besides the first line classical treatment (chemotherapy, radiotherapy), CIK therapy continues to evolve in parallel with other immunotherapies by synergising with advanced immunologic approaches like antibody therapy (monoclonal antibody, bispecific monoclonal antibodies, bispecific T-cell engager (BiTE) antibody), checkpoint/epigenetic inhibitors, chimeric antigen receptor T cell CAR-T cell therapy, allogeneic hematopoietic cell transplantation (alloHCT), Antibody-drug conjugates (ADCs), combination with cytokine inhibitors and recently proposed CIK/DC–CIK cells with nanomaterials [7–9]. Although mild graft-versus-host disease (GVHD) is still a concern in a subset of patients treated with CIK cells, experience from previous clinical trials suggests that these are largely manageable [10]. Notwithstanding the fact that the clinical landscape is at an interesting phase, as several drug targets are currently available and trials are ongoing, recurrence (relapse) and unresponsiveness to the treatment is a general problem. Up to greater extent, cancer heterogeneity is accountable for this intrinsic or acquired resistance, resulting into treatment failure (resistance or relapse) after therapy in the majority of patients. Like other cancer immunotherapies, the complete success of CIK cell therapy is also hindered by this aforementioned factor. Nevertheless, being a heterogeneous cell population, CIK cells possess the ability to target and kill heterogeneous tumor cells.

Since most of the clinical trials are still in an early phase, there are some concerns that require open discussion, such as 1) how comparable are the toxic effects of CIK cells to those of CAR T cells, NK cells and other immune cells after infusion, 2) how can we minimize the additional supportive care that is still required after CIK therapy in order to avoid prolonged treatment, 3) certainly CIK therapy is safe for a subset of patients, is that enough to consider it advantageous over other immunotherapeutic approaches, 4) exactly what key determinants (genetic, epigenetic, immunological, etc.) are required for the crosstalk with CIK cells to create in the inter/intra-individual cancer microenvironment, 5) can we identify (initially in preclinical models) the underlying factors that alter the effects of peripheral blood-derived CIK cells (PB-CIK) compared to cord blood-derived CIK cells (CB-CIK) in the clinical trials, 6) how can we “generalize” this approach by extending it to multiple centers.

Considering the results of the studies conducted internationally, it is reasonable to conclude that CIK therapy has great potential in this rapidly evolving landscape of cancer therapies. Therefore, optimizing CIK therapy in combination with other therapeutic modalities, primarily in a complementary manner (rather than competitive) will be a key to combat cancer in clinical practice and to prolong the survival rate of cancer patients.

## Conclusion

CIK therapy has great potential and its optimization (comparison of results with those of other therapies) to define combining options with other therapeutic modalities can improve the cancer scenario in clinics.

## Data Availability

Not applicable.

## Abbreviations

CIK: Cytokine-induced killer cells
DC: Dendritic cell
NKG2D: Natural killer group 2 member D
alloHCT: Allogeneic hematopoietic cell transplantation
ADCs: Antibody-drug conjugates
ADCC: Antibody-dependent cellular cytotoxicity
GVHD: Graft-versus-host disease
